# Mapping and Functional Dissection of the Rumpless Trait in Piao Chicken Identifies a Causal Loss of Function Mutation in the Novel Gene *Rum*

**DOI:** 10.1093/molbev/msad273

**Published:** 2023-12-09

**Authors:** Ying Guo, Jing Tian, Chi Song, Wei Han, Chunhong Zhu, Huifang Li, Shuangjie Zhang, Kuanwei Chen, Ning Li, Örjan Carlborg, Xiaoxiang Hu

**Affiliations:** State Key Laboratory of Animal Biotech Breeding, China Agricultural University, Beijing CN-100193, China; National Engineering Laboratory for Animal Breeding, China Agricultural University, Beijing CN-100193, China; Yazhouwan National Laboratory, Sanya CN-572024, China; Department of Medical Biochemistry and Microbiology, Uppsala University, Uppsala SE-751 23, Sweden; State Key Laboratory of Animal Biotech Breeding, China Agricultural University, Beijing CN-100193, China; National Engineering Laboratory for Animal Breeding, China Agricultural University, Beijing CN-100193, China; Inner Mongolia Academy of Agricultural & Animal Husbandry Sciences, Hohhot CN-010031, China; National Chickens Genetic Resources, Jiangsu Institute of Poultry Science, Yangzhou CN-225125, Jiangsu, China; National Chickens Genetic Resources, Jiangsu Institute of Poultry Science, Yangzhou CN-225125, Jiangsu, China; National Chickens Genetic Resources, Jiangsu Institute of Poultry Science, Yangzhou CN-225125, Jiangsu, China; National Chickens Genetic Resources, Jiangsu Institute of Poultry Science, Yangzhou CN-225125, Jiangsu, China; National Chickens Genetic Resources, Jiangsu Institute of Poultry Science, Yangzhou CN-225125, Jiangsu, China; National Chickens Genetic Resources, Jiangsu Institute of Poultry Science, Yangzhou CN-225125, Jiangsu, China; State Key Laboratory of Animal Biotech Breeding, China Agricultural University, Beijing CN-100193, China; National Engineering Laboratory for Animal Breeding, China Agricultural University, Beijing CN-100193, China; Department of Medical Biochemistry and Microbiology, Uppsala University, Uppsala SE-751 23, Sweden; State Key Laboratory of Animal Biotech Breeding, China Agricultural University, Beijing CN-100193, China; National Engineering Laboratory for Animal Breeding, China Agricultural University, Beijing CN-100193, China

**Keywords:** rumpless, Piao chicken, genetic mapping, functional analyses, somitogenesis

## Abstract

Rumpless chickens exhibit an abnormality in their tail development. The genetics and biology of this trait has been studied for decades to illustrate a broad variation in both the types of inheritance and the severity in the developmental defects of the tail. In this study, we created a backcross pedigree by intercrossing Piao (rumpless) with Xianju (normal) to investigate the genetic mechanisms and molecular basis of the rumpless trait in Piao chicken. Through genome-wide association and linkage analyses, the candidate region was fine-mapped to 798.5 kb (chromosome 2: 86.9 to 87.7 Mb). Whole-genome sequencing analyses identified a single variant, a 4.2 kb deletion, which was completely associated with the rumpless phenotype. Explorations of the expression data identified a novel causative gene, *Rum*, that produced a long, intronless transcript across the deletion. The expression of *Rum* is embryo-specific, and it regulates the expression of *MSGN1*, a key factor in regulating T-box transcription factors required for mesoderm formation and differentiation. These results provide genetic and molecular experimental evidence for a novel mechanism regulating tail development in chicken and report the likely causal mutation for the tail abnormity in the Piao chicken. The novel regulatory gene, *Rum*, will, due to its role in fundamental embryo development, be of interest for further explorations of a potential role in tail and skeletal development also in other vertebrates.

## Introduction

**Fig. 1.**
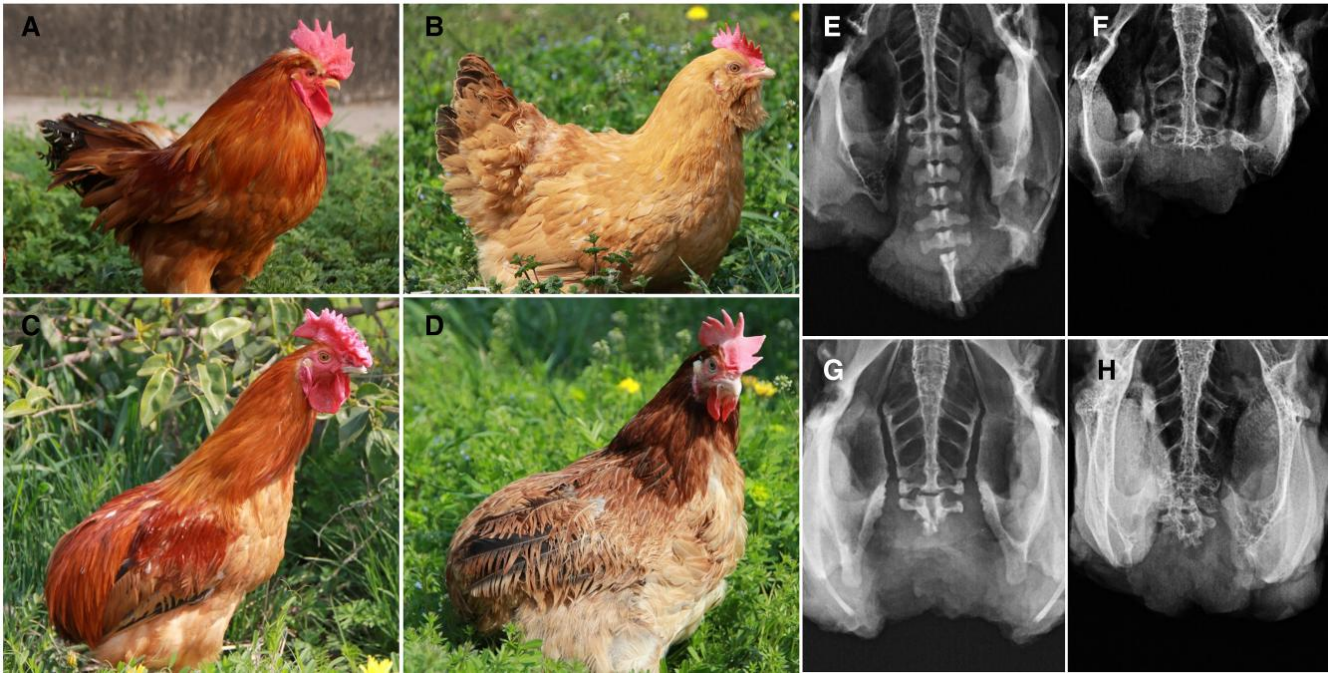
The rumpless Piao chicken. The WT cock (A), hen (B), Piao rumpless cock (C), and hen (D) are pictured, together with X-rays showing the tailbones of a WT (E) and 3 degrees of the rumpless trait (F, G, H) displayed in the Piao breed.

Abnormal development of the skeleton is uncommon in chicken; most reported cases in the poultry industry are induced by nutrition or environment (Thorp 1994). Although genetic disorders of the skeleton are rare, they do from time to time arise in breeding flocks. The bone anomaly “Rumplessness,” a lack of normal tail, has over the years been introduced in a number of chicken breeds around the world including Piao (Chinese), Araucana (American), Manx Rumpy (UK), Drentse Bolstaart (Dutch), Kaulhuhn (Germany), Barbu d'Everberg (Belgian), and Ardenner Bolstaart (Belgian). Here, we study this trait in a Chinese local breed, Piao. The morphology of the caudal region of the rumpless Piao includes a lack of the fleshy rump, the uropygial (oil) gland, the tail feathers, pygostyle, and free caudal vertebrae that are irregularly fused in some cases (Fig. 1 and supplementary GIF files, Supplementary Material online).

The genetics of the rumpless trait has been studied for decades, starting with studies focusing on the basic inheritance (Dunn 1925; Dunn and Landauer 1934; Dunn and Landauer 1936; Landauer 1945) and development (Zwilling 1942) of the trait in different populations, finding it to be diverse across populations (Dunn 1925). Both dominant and recessive inheritance has been reported (Dunn 1925; Landauer 1945), often with variable extent of tail loss (Dunn 1925; Dunn and Landauer 1936). More recently, researchers have utilized genome resequencing to characterize several rumpless stocks to study the molecular genetic basis of the trait (Freese et al. 2014; Jeong et al. 2016; Wang et al. 2017; Wang et al. 2018; Noorai et al. 2019). No causative mutations have been identified, but strong association signals have been reported on chromosome 2 in the Araucana breed (Freese et al. 2014; Noorai et al. 2019).

Early studies on mapping of the rumplessness trait in Piao through population comparisons have identified the associated region on chromosome 2 (Wang et al. 2021) and the candidate mutation, which is a structural variant (Zhang et al. 2022). While the specific role of this structural variation (SV) and its exact breakpoints weren’t explored in depth, the causal gene and the genetic mechanisms that underpin the trait's formation also remain undefined. Although genes expression was explored in Wang's study (Wang et al. 2021), the stages (E7 to E9) are not pivotal for the formation of the tail somite. Hence, the causal gene together with its critical functions and expression patterns might have been overlooked.

In the current study, we scored the somite numbers for rumpless Piao embryos to characterize the morphological effects of the Piao rumpless mutation. By crossbreeding the rumpless Piao with the normally tailed Xianju breed, we investigated the genetic basis of this skeletal defect. Our comprehensive variant screening within the targeted region revealed that the single nucleotide polymorphisms (SNPs) were not the contributing factors. A notable 4.2 kb deletion aligned with prior research by Zhang et al. (Zhang et al. 2022) was confirmed to correlate fully with rumplessness in Piao chickens. Furthermore, our findings highlighted the presence of a novel gene, *Rum*, which demonstrates exclusive expression during embryonic stages. *Rum* serves as a pivotal regulator, exerting influence over the expression of *MSGN1* and thereby contributing to the development of the rumpless phenotype in the Piao chicken during the mesoderm formation and differentiation.

## Results

We studied both the physiology and genetics of the rumpless trait in the Piao chicken breed and the results are described in detail below.

### Physiological Characterization of the Rumpless Trait in the Piao Chicken Breed

#### Variation in Tail Lengths Quantified Using X-Ray Analyses

In total, *n* = 573 backcross chickens were bred across generations BC1 to BC6 (Table 1). A variation in tail lengths was observed among rumpless chickens (Fig. 1D to F) both in the BC1 to BC6 generations, as well as in the offspring of an intercross between BC1 chickens (BC1SM). To explore and quantify the physiological basis of this, we used X-ray analyses to score the phenotype in *n* = 194 chickens from the BC2 to BC4 generations and the BC1SM. Most of the rumpless chickens still had between 1 and 5 free caudal vertebra (supplementary table S1, Supplementary Material online), while only 6 samples had lost the pygostyle and entire caudal vertebra. No significant differences in the mean numbers of caudal vertebra number were found in the rumpless chickens of the different generations (BC2 = 2.4, BC3 = 1.8, BC4 = 2.2, and BC1SM = 1.7; nonsignificant in a Wilcoxon signed-rank test).

**Table 1:** Segregation of the rumpless trait in the Piao × Xianju backcross population

Generation	Normal		Rumpless		Total	Rumpless (%)
	Female	Male	Female	Male		
BC1	4	5	2	6	17	47.1%
BC2	35	34	32	28	129	46.5%
BC3	31	44	33	52	160	53.1%
BC4	37	28	35	38	138	52.9%
BC5	17	19	12	28	76	52.6%
BC6	17	9	17	10	53	50.9%
Total	141	139	131	162	573	51.1%

### Reduced Numbers of Somites in Rumpless Embryos

**Fig. 2.**
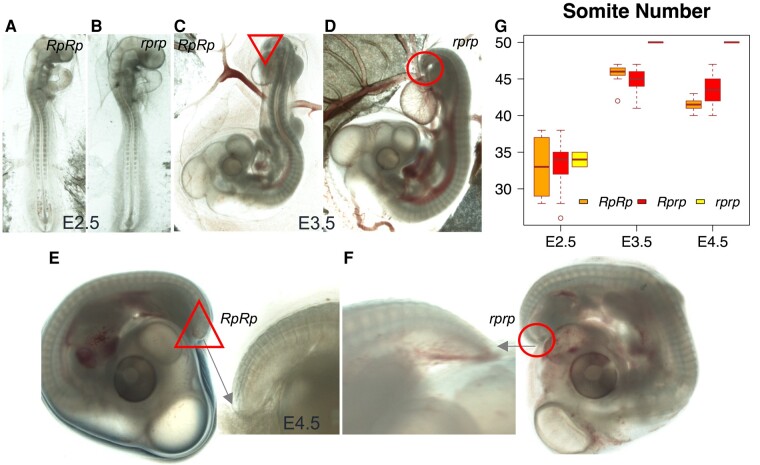
Evaluation of the somite number at different incubation stages. Somitogenesis was examined in Piao Chicken from embryonal stages E2.5 to E4.5 (A to F). The rumpless embryos showing reduced numbers of somite pairs are presented on the left. G) A summary of somite numbers in *RpRp*, *Rprp*, and *rprp* embryos from days E2.5 to E4.5.

Somites are important during embryo development where they, in vertebrates, develop into cartilage, skeletal muscle, and bone (Middleditch and Oliver 2005). In chicken, the somite number has been previously reported to range from 47 to 55 pairs (Bellairs 1986), but this number is reduced in rumpless chickens (Zwilling 1942). In our study, we scored the number of somites in 162 embryos from Bc and pure Piao populations at different developmental stages (Fig. 2, Table 5). The results showed that somites had extended to the tip of the tail around E3.5 to E4, and by that time, the wild-type (WT) embryos had a total of 50 pairs of somites, while the rumpless individuals typically had <47 pairs of somites (with an average reduction of 6 pairs; Fig. 2G). The phenotypic difference is also visible after E3.5 as a shorter and blunter tip of the tail in the rumpless individuals compared with normal embryos (Fig. 2C to F).

### Genetic Analyses of the Rumpless Trait

#### Piao Rumpless Is an Autosomal Dominant Trait

To generate the backcross resource population, 1 rumpless male Piao chicken was first mated with several normal-tailed Xianju females to generate the BC1 generation. Then, 1 rumpless male was selected in each Bc generation for backcrossing to Xianju females for breeding of generations BC2 to BC6. The ratio of rumpless to normal birds was close to 1:1 in all backcross generations (Table 1), with no significant deviation from this when tested across all backcross chickens using a Chi-square test (χ^2^ = 0.29494; *P* value = 0.5871). The overall male:female ratio in the backcross population was also close to 1:1 (χ^2^ = 1.4677, *P* value = 0.2257). These results demonstrate the autosomal dominant inheritance of the rumpless trait in the Piao chicken breed.

#### Is Lack of Fixation of the Rumpless Trait in Piao a Result of the Mutation Being Partially Lethal when in Homozygote Form?

Despite more than 15 yr of selective breeding based on the phenotype, the rumpless trait has not yet been fixed in the Piao breed. We therefore tested the hypothesis of this being due to partial homozygote lethality. To test this, we first used the collected data from the intercross between heterozygous (*Rprp × Rprp*) BC1 individuals. A total of 34 BC1SM offspring were available, with genotype ratios *RpRp*:*Rprp*:*rprp* of 4:21:9 which did not significantly deviate from the expected 1:2:1 ratio (χ^2^ = 1.5348, *P* value = 0.2154). However, the small data set may have limited statistical power. Therefore, we conducted an independent test using embryos from purebred Piao collected at stages E2.5 to E6.5. The eggs were randomly selected, and the sample was assumed to be representative of the Piao population. The numbers of individuals for the 3 genotypes (*RpRp*/*Rprp*/*rprp*) in these samples were 24:43:9, with estimated allele frequencies for *Rp* and *rp* being *P* = 0.6 and *q* = 0.4, respectively. No significant deviation from the expected Hardy–Weinberg equilibrium (HWE) genotype frequencies was found for *RpRp* (χ^2^ = 0.46713, *P* value = 0.4943). Thus, we found no statistical evidence to support the hypothesis that the rumpless mutation causes partial lethality in homozygotes.

#### GWAS Identifies a Region on Chromosome 2 Associated with the Piao Rumpless Trait

**Fig. 3.**
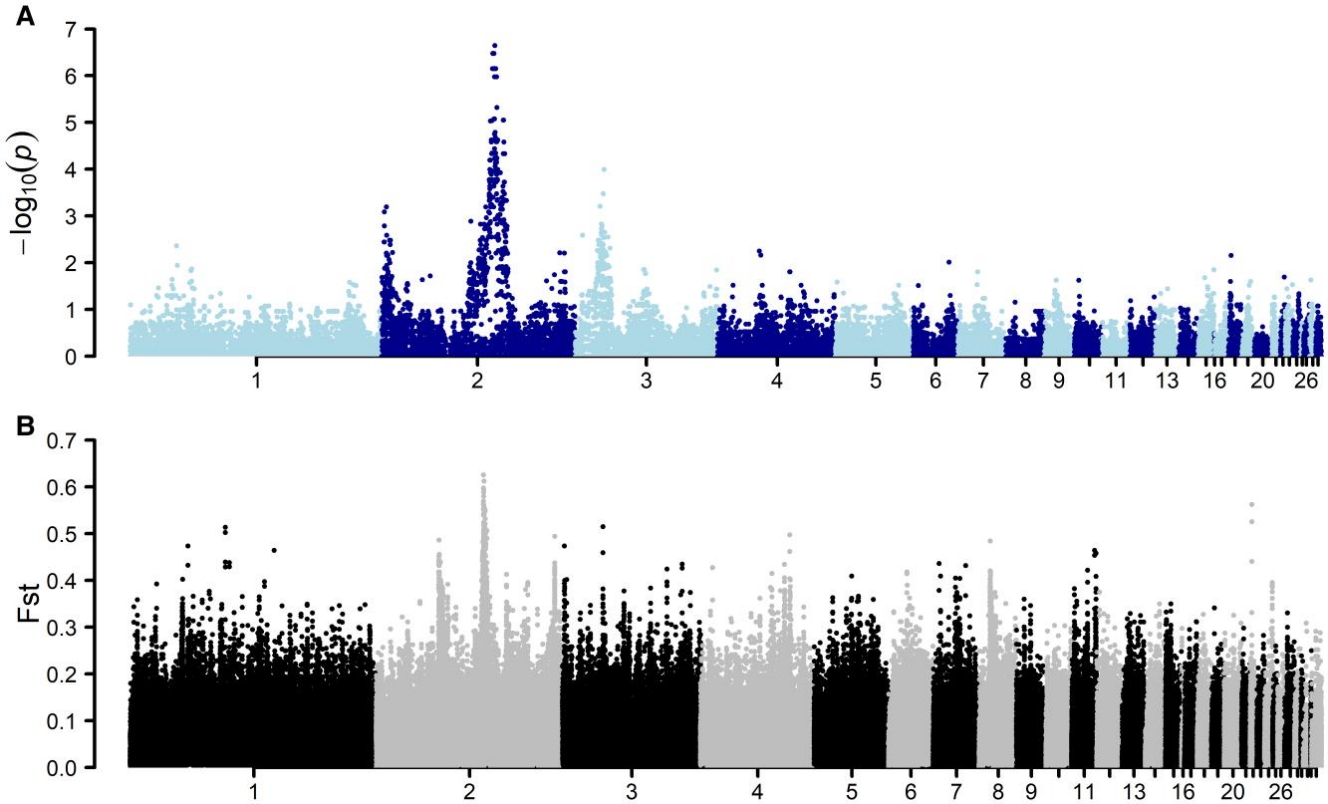
A significant association signal on chromosome 2 to rumplessness in the Piao chicken breed. A) GWAS using 33 backcross samples showed a strong association with rumplessness on chromosome 2. B) Genome-wide pairwise comparison of the Fst using rumpless (Piao and Bc) and normal samples with a sliding window size of 5 kb. The highest peak here was also observed on chromosome 2 in the same region as the GWAS association.

To explore the genome-wide genetic basis of the rumpless trait in Piao, individuals from BC1 and BC2 (*n* = 16 cases and *n* = 17 controls) were whole-genome genotyped using the chicken 60 K SNP array. A genome-wide association analysis (GWAS; Fig. 3A) in this data identified a strong signal on chromosome 2 (∼86.2 to 88.7 Mb). This region overlapped that earlier reported for the rumpless trait in the Araucana and Piao chicken breed (Noorai et al. 2012; Zhang et al. 2022).

#### Population Genomics Analyses to Detect Selection Signals in Rumpless Chicken Stocks

**Fig. 4.**
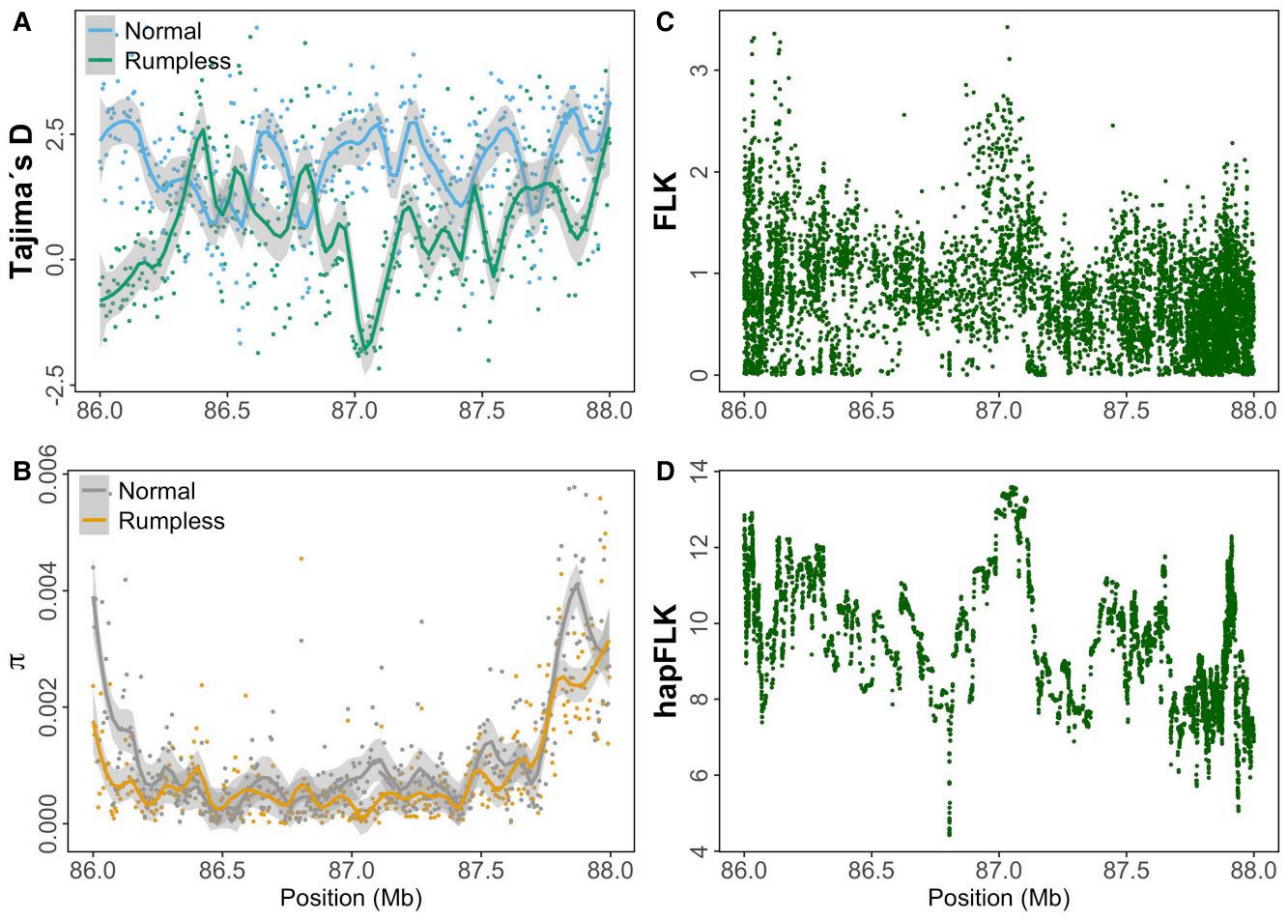
The selective sweep signatures in the peak region on chromosome 2. The average Tajima’s D (A) and nucleotide diversity (π, B) for sliding window sizes of 5 kb in rumpless Piao and normal chickens. The FLK (C) and hapFLK (D) analyses also show a selection signature in the same target region. Results are obtained using individual genotyping data from the rumpless Piao and 8 normal stocks.

The population-level divergence of the rumpless Piao was explored by comparing sequences from this breed with those of 8 WT chicken populations including Black Minorca, Langshan, Dominique, Black Cochin, Buff Cochin, Partridge Cochin, Java, and Light Brahma (Table 4), in which the data were obtained from one of our previous studies (Guo et al. 2019). The result showed a significant difference in the genomic region from 86.2 to 86.9 Mb on chromosome 2, as indicated by fixation index (Fst) (Fig. 3B). Additionally, the study identified substantial decreases in Tajima's *D*, concurrent with declines in nucleotide diversity (π), and observed peaks in FLK/hapFLK analyses. These findings collectively suggest a selective sweep in the rumpless Piao chicken population within this region (Fig. 4).

#### Linkage and Identity-by-Descent Mapping–Based Fine-Mapping of the Piao Rumpless-Associated Region on Chromosome 2

*Plink* (Chang et al. 2015) was used to perform identity-by-descent mapping (IBD)-based fine-mapping in the backcross population using the 60 K SNP array data from purebred Piao (*n* = 32), BC1 (*n* = 1), and BC2 (*n* = 33) samples. A 2.55 Mb IBD segment (chromosome 2: 86,242,890 to 88,788,820 bp) was detected as shared by all the Piao rumpless chickens. Additional genotyping was next performed across this 2.55 Mb region using both *Sequenom MassARRAY* and *Fluidigm SNP Type* in samples from the Piao × Xianju backcross, purebred Piao, purebred Xianju, and several normal breeds. A total of 34 SNPs (21 from *Sequenom* and 13 from *Fluidigm*) were found to segregate and were used for building a linkage map in BC2. Next, a linkage analysis using samples from the BC2 family identified a 798.5 kb region (chromosome 2: 86,902,825 to 87,701,351 bp) in complete linkage with the rumpless trait.

#### The Rumpless Phenotype in the Piao Breed Is Likely Due to a Novel Genetic Variation on Chromosome 2

**Fig. 5.**
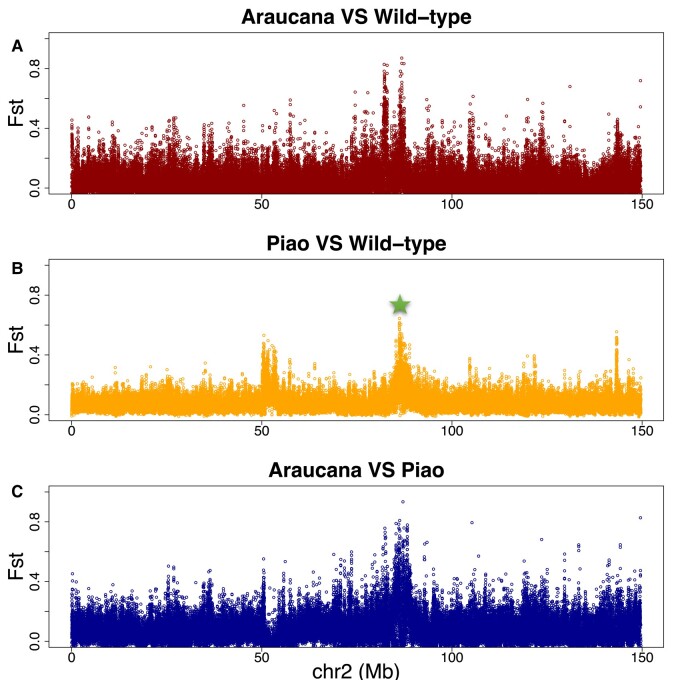
Analyses of the selection on chromosome 2 among rumpless Araucana, rumpless Piao, and 8 WT chicken populations. The target region for Piao chicken is pointed out with the green star. Different selection signals can be found between Araucana/WT (A) and Piao/WT (B) along the chromosome and also between Araucana and Piao at the target interval (C).

The region associated with the rumpless phenotype in the Piao breed was located on chromosome 2 and very close to the one earlier reported to be associated with the rumpless phenotype in an American chicken breed called Araucana (Noorai et al. 2012; Freese et al. 2014; Noorai et al. 2019). Population genomics analyses of Piao, Araucana, and multiple other populations with normal tail identify selection for both rumpless breeds in the same region of chromosome 2 (Fig. 5A and B). These rumpless breeds are, however, divergent from one another in this target region (Fig. 5C). Further, the 125 kb (86,456,876 to 86,582,539 bp, Galgal6) region reported in the Araucana chicken (Freese et al. 2014) did not overlap with the ultimately fine-mapped region reported here in Piao chicken (86,902,825 to 87,70,1351 bp). The 2 candidate SNPs (86,475,370 and 86,575,283 bp, Galgal6) previously identified on chromosome 2 in Araucana (Freese et al. 2014) were analyzed in our data set. The findings indicate that all rumpless Piao chickens exclusively possess the reference alleles. These results suggest that the rumpless trait in Piao and Araucana likely originated from different causal mutations.

#### Screening Whole-Genome Sequence Data for Candidate Functional Variants Determining the Piao Rumpless Phenotype

All scored SNPs in the final 798.5 kb target region obtained from whole-genome resequencing were explored as potential functional candidate mutations for the trait. For this autosomal dominant trait with complete penetrance, a functional polymorphism should fulfill the criteria of (i) being heterozygous in all the rumpless samples of the backcross family and (ii) being homozygous for the reference allele in normal samples. In total, 96 of 2,181 SNPs in this region were heterozygous in the rumpless backcross offspring. Of these, 85 SNPs were excluded since the alternative variants were also presented in normal samples and the remaining 11 SNPs were excluded since homozygous for the reference alleles were found in purebred Piao rumpless individuals.

**Fig. 6.**
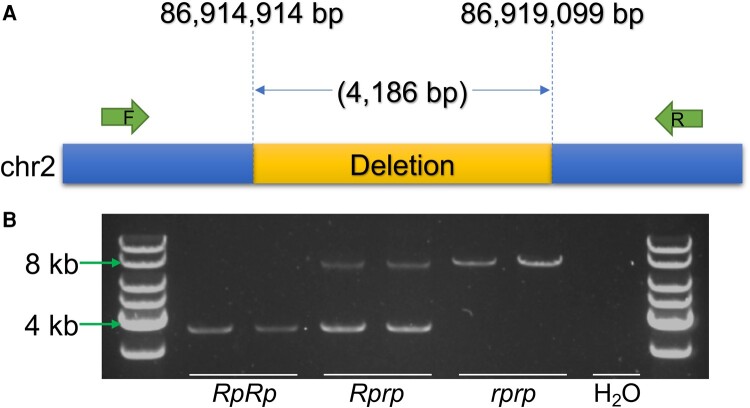
A functional candidate 4.2 kb deletion on chromosome 2 that is completely associated with the rumpless trait in the Piao chicken breed. A) The breakpoints of the deletion and location of the primers used for detection. B) Validation of the deletion using PCR amplification in *RpRp*, *Rprp*, and *rprp* chickens.

Since no individual functional candidate SNPs were identified, the candidate region was next screened for SVs. The *BreakDancer-1.3.6* (Fan et al. 2014) software was used to analyze sequence data from 128 chickens (50 rumpless Piao and 78 normal from the abovementioned 8 WT populations) and identified a deletion (chromosome 2: 86,914,972 to 86,918,969) in the 798.5 kb target region. In this analysis, it was reported to be present in 45 of 50 evaluated rumpless chickens. However, as the depth of sequencing was not sufficiently high to expect detection of all deletions, validation of the deletion was also performed using polymerase chain reaction (PCR). This confirmed the deletion in the 5 rumpless samples that did not show it in the initial *BreakDancer* analysis and also in 384 additional samples (supplementary table S3, Supplementary Material online) included to further strengthen the association of the deletion with the rumpless trait. The size of the amplification segment in heterozygous individuals showed the length of the deletion to be around 4 kb. Sequencing of the PCR products was performed, and this identified the breakpoints for the SV as 86,914,914 and 86,919,099 bp resulting in a size of the deletion of 4,186 bp (Fig. 6). The estimation for age of the deletion was carried out using *GEVA* (Albers and McVean 2020) on the variants within the 798.5 kb region. The deletion was treated as a SNP with its genotype identified as the genotype of the SNP. The result suggests that the rumpless mutation emerged around 1,475 years ago. Additionally, we verified the deletion discovered in Piao using data (mean depth 11.8×) from Araucana chickens (Noorai et al. 2019). Our analysis revealed the absence of this deletion in rumpless Araucana chickens.

### Identifying the Regulatory Mechanism of the Rumpless Mutation by Integrated Functional Genomic Analyses

#### Functional Explorations of the Potential Effects of the Deletion

The sequence of the 4.2 kb deletion, which was completely linked to the rumplessness in Piao, exhibits high conservation in birds, as demonstrated by *multiz* alignments of 77 vertebrates (data from University of California Santa Cruz [UCSC]; supplementary fig. S1, Supplementary Material online). The exception to this conservation is the insertion of the CR1-F element. This observation suggests that the CR1-F element may have emerged after the divergence of the Gallus genus. Within the deleted segment, a putative transcription start site (TSS) was detected, supported by 52 reads from the 5′ end of CAGE in the FANTOM5 data set (Abugessaisa et al. 2016; Lizio et al. 2017). Furthermore, a TATA box (*P* value: 7.1000e^−03^) motif was identified using the *SSA* motif discovery tools (Ambrosini et al. 2003) based on characteristics and position of the sequence. Additionally, an enhancer (chromosome 2: 86,917,199 to 86,917,618 bp) was predicted by the *EnhancerAtlas 2.0* (Gao and Qian 2020) in DT40 cells. We explored the chromatin topology of the deletion region using data from previous work (Fishman et al. 2019; Foissac et al. 2019) and found that the target site contains a topology associated domain (TAD).

In Araucana (Freese et al. 2014), it was earlier reported that misexpression of *IRX1* and *IRX2* was a regulator of the rumpless phenotype. We therefore explored this hypothesis in more detail by quantifying the expression of *IRX1* and *IRX2* in the tailbud of rumpless Piao embryos at stages E3.5 (HH21), E4.5 (HH25), and E5.5 (HH28). These developmental stages were chosen as the somites will extend to tip of tail starting at E3.5 (Hamburger and Hamilton 1951). Expression of *IRX1* and *IRX2* was observed in both WT and rumpless embryos (supplementary fig. S2, Supplementary Material online). This is consistent with the in situ hybridization results from GEISHA (Wang et al. 2017), a chicken embryo gene expression database, where expression of both *IRX1* and *IRX2* is reported in the mesoderm and later in the paraxial mesoderm including *IRX1* expression in the tailbud at HH18 (E3 days) (http://geisha.arizona.edu/geisha/photo.jsp?url_path=/geisha/photos/&id=29617). Our results thus, in contradiction to the earlier research on the rumpless phenotype in the Araucana breed, do not find ectopic expression of *IRX1* and *IRX2* to be the driver of the rumpless phenotype in Piao.

#### Discovery of a Novel Transcript in the Deleted Segment of the Rumpless Piao Allele

**Fig. 7.**
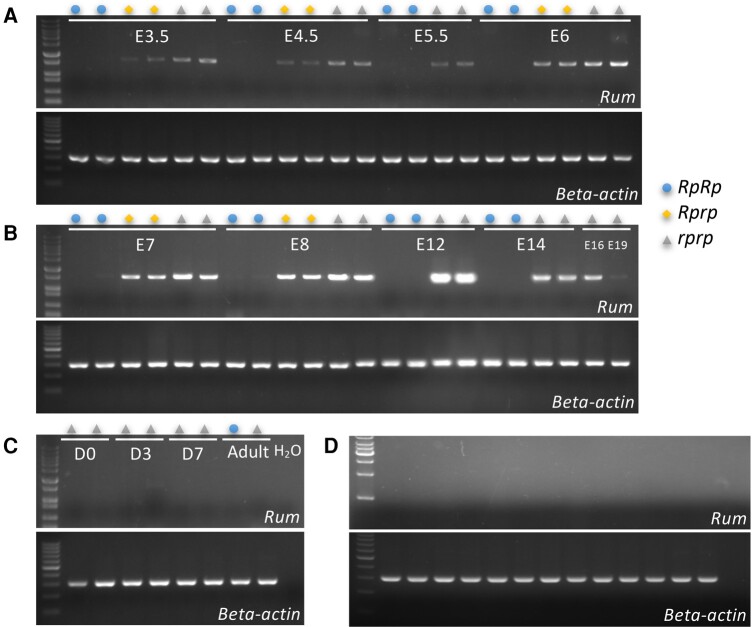
Expression analyses of this novel transcript *Rum*. The expression of *Rum* is detected in the tail tissues of embryos (A and B), as well as in chicks and adults (C). Additionally, expression was also detected in WT tissues (D). The samples were analyzed in the following order, from left to right: heart, liver, spleen, kidney, duodenum, jejunum, ileum, pectoralis muscle, thigh muscle, proventriculus, gizzard, thoracic cartilage, leg cartilage, and H_2_O.

Next, we explored the hypothesis that the deletion in the rumpless Piao allele either disrupted, or resulted in the loss of, a novel expressed sequence located within or overlapping with it. Open reading frame (ORF) prediction using ORFfinder (https://www.ncbi.nlm.nih.gov/orffinder/) identified 1 1,074 bp long ORF overlapping with a CR1 element (CR1-F, divergence = 3.2%, chromosome 2: 86,916,993 to 86,918,203 bp). The transcript was validated using reverse transcription (RT)-PCR analyses in tail tissue from E4.5, and the full-length fragment was revealed by “walking through” it from both ends using cDNA amplification (supplementary fig. S3, Supplementary Material online). This novel gene, which we denote *Rum*, is ∼22 kb long (chromosome 2: 86,902,316 to 86,924,585 bp) and contains the entire deleted segment of the Piao allele. Expression profiling of the *Rum* gene showed that it was limited to the embryo (Fig. 7) and that expression was lost in *RpRp* and reduced in *Rprp* in chicken (Fig. 7A and B).

The expression of *Rum* was also assessed in other species including mouse, Chinese hamster, Campbell's hamster, and *Poecilia parae*, using expression data from the tail tissues of embryos. The data were obtained from publicly available data sets on the National Center for Biotechnology Information (NCBI), with the accession numbers PRJNA798668 and PRJDB9148. We aligned all reads from these samples to the target genomic region (*Rum* is an intronless gene). Notably, we encountered a challenge when attempting to map reads to the *Rum* gene region in species other than chicken which indicates absent expression of *Rum* in the other vertebrates. We also analyzed *Rum* gene expression in embryonic tail tissues from ducks (E6, *n* = 2) and pigeons (E2.5, E3.5, E4.5, *n* = 2, 2, 2). The analysis revealed no evidence of *Rum* gene expression in either of these bird species.

#### Transcriptome Analysis to Identify Genes Regulated by *Rum*

RNA-seq analysis was used to detect differentially expressed genes that could potentially be regulated by *Rum*. For this, libraries were prepared for samples from all 3 genotypes obtained at E3.5 and E4.5 and sequenced using both the Pacbio RS II (*RpRp*, *Rprp*, *rprp*; *n* = 2, 2, 2) and Illumina NovaSeq 6000 systems (*RpRp*, *Rprp*, *rprp*; *n* = 6, 6, 6). Genes that were significantly differentially expressed at the 2 timepoints between genotypes are shown in supplementary table S7, Supplementary Material online. Read coverages for the candidate genes were manually verified using *IGV* (Robinson et al. 2011). The result revealed the presence of a long *MSGN1* transcript exclusively in the *rprp* samples, indicating differential transcript expression between genotypes. To explore this further, RACE was utilized to obtain complete *MSGN1* transcript(s) in *rprp* individuals. The results confirmed the existence of 2 transcripts for *MSGN1* in WT chickens, with the short transcript being previously annotated and the novel long transcript being included in the most recent annotation release (ID: 106). This long transcript spans 3,414 bp, starting at position 100,204,512 bp and ending at 100,207,925 bp. Primers were designed to detect the expression of both the long-specific and common regions of *MSGN1* in all genotypes using RT-PCR. The results revealed that the expression of the long transcript of *MSGN1* was barely detectable in rumpless samples (supplementary fig. S4, Supplementary Material online).

**Fig. 8.**
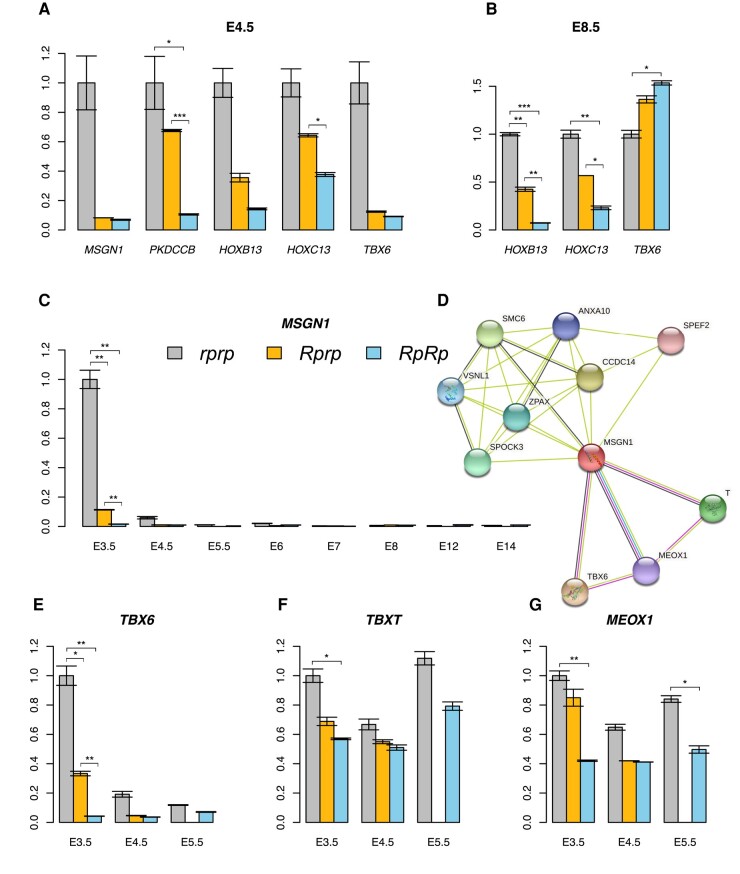
Expression analyses of differentially expressed genes in the tail tissue of embryos. The relative expression of significantly differentially expressed genes was analyzed at E4.5 (A) and E8.5 (B), as well as the long transcript of *MSGN1* gene from E3.5 to E14 (C). The predicted protein–protein interaction network of MSGN1 (D). The relative expression of *TBX6* (E), *TBXT* (F), and *MEOX1* (G) was also evaluated at E3.5, E4.5, and E5.5. The vertical axis in each figure indicates the relative expression of each gene, normalized to the value of *rprp* at E3.5, which was defined as 1. Error bars in the figures represent the standard deviation. ***P < 0.001, **P < 0.01, *P < 0.05.

Genes that showed differential expression at both E3.5 and E4.5 were further investigated, along with genes that displayed potential functional relevance based on literature studies but were only differentially expressed at 1 timepoint. Samples from all 3 genotypes were used (with 3 biological repeats each) at E4.5 and E8.5. Significant differentially expressed genes in this analysis are shown in Fig. 8A and B. The expression of the long alternative transcript of *MSGN1* that was barely detectable in *RpRp*, significantly differentially expressed between *Rprp* and *rprp* at E3.5, and almost absent in all genotypic samples starts from E6 (Fig. 8C). The protein association network of MSGN1, inferred using *STRING*, highlighted the involvement of genes related to tail development including *T(BXT)*, *TBX6*, and *MEOX1* (Fig. 8D). Of these 3 genes, only *TBX6* (Fig. 8E and F) exhibited a similar expression pattern to *MSGN1*, with its expression being deregulated in *Rp-* at E3.5 but returning to normal levels (compared with WT) by around E5.5 (Fig. 8E) which was further verified at E8.5 (Fig. 8B).

## Discussion

### Rumplessness in Chickens: Impacts on Fertility, Embryonic Mortality, and Hatchability in the Piao Breed

The rumpless trait in chicken is characterized by the absence of the last few vertebrae and the tail, as well as abnormal vertebral fusion in caudal region (Fig. 1). Building on the research findings of Dunn and Landauer (Dunn and Landauer 1934), it is evident that rumpless fowls exhibit reduced fertility when compared with normal ones. The primary contributing factor to this decline in fertility is likely the altered morphology, which leads to mating difficulties. Although rumpless fowls may exhibit slightly lower viability compared with normal fowls, it is important to note that the homozygote is not lethal as addressed by Dunn and Landauer (Dunn and Landauer 1934).

In the Piao chicken, artificial fertilization led to a significant fertility boost, with rates reaching 89.4%. Most of the deceased embryos were found during the 18th to 21st days of incubation, a phenomenon common in other chicken breeds (Payne 1919; Romanoff 1949). Various factors contribute to this late-stage embryonic mortality, including infection, disease, improper temperature and humidity, inadequate ventilation, and occasionally, genetic reasons (Romanoff 1949; Noiva et al. 2014). Data from National Gene Bank for Local Chicken Breeds (Jiangsu, China) reveal that when Piao is incubated alongside other Chinese local breeds under identical conditions, the hatchability rate decreases to 81.4%. However, by incubating Piao separately at a slightly higher temperature, hatchability can be improved, reaching up to 90%. Compared with other local Chinese chicken breeds, we did not note a higher posthatching mortality rate in the Piao breed.

### The Redundancy in Genetic Pathways Converging to Yield the Same Rumplessness Phenotype in Piao and Araucana Chicken Breeds

Here, we characterized, mapped, fine-mapped, and evaluated the population genomics signature of selection for the rumpless trait in the Piao chicken breed. A long rumpless haplotype was identified, visible as a long run of homozygosity. This finding aligns with the hypothesis that the Piao breed has experienced a bottleneck, undergone intensive selection, and been bred in a small population size, which is consistent with the reports from a survey in 2009 (Chen et al. 2011).

The GWAS-associated region broadly overlapped with the one earlier reported as associated with the rumpless trait in the American Araucana breed (Noorai et al. 2012; Freese et al. 2014; Noorai et al. 2019). It is noteworthy that Araucana and Piao are genetically distinct breeds with no recent common ancestry. Piao is an old, local Chinese chicken breed originating from, and still mainly bred, in the Yunnan province. The American Araucana originates from Chile (Ekarius 2007) and was later imported to the United States, where it was standardized in 1976 (https://en.wikipedia.org/wiki/Araucana#cite_note-nestor-5). The overall appearance and major phenotypes of the rumpless Araucana and the Piao are also different, including, for example, body size, ear tuft, and egg color. All this is consistent with the population genomics results illustrating the diversity between Piao and Araucana both genome-wide and in the associated region on chromosome 2.

In our current study, we have confirmed the presence of divergent causal mutations specific to these 2 breeds. Consequently, if these distinct genetic backgrounds result in the manifestation of the same phenotype, it implies that nature has evolved alternative genetic solutions to achieve the desired trait. This redundancy in genetic pathways converging to yield a common phenotype serves as a remarkable example of evolutionary versatility, prompting profound inquiries into the underlying molecular and developmental mechanisms. Our investigations in both Araucana (Freese et al. 2014) and Piao have uncovered the involvement of the *TBX6* gene in both genetic pathways, emphasizing the potential for convergent rumplessness to share a common downstream network.

### Analyses of Candidate Genes at the Target Region Reveal Indistinctive Expression for Both IRX1 and IRX2

In the fine-mapped region, the strongest candidate genes based on earlier functional and genetic work is *IRX1* and *IRX2*, both from the Iroquois homeobox factor family. Genes in this family are 2 clusters (A and B) of 3 transcription factors that are conserved in vertebrates with similar genomic organization (Gómez-Skarmeta and Modolell 2002). The Iroquois genes are necessary for neural (Gómez-Skarmeta and Modolell 2002) and limb (McDonald et al. 2010) development and also pattern formation during early and late development (Gómez-Skarmeta and Modolell 2002). Earlier research on *IRX1* and *IRX2* in chicken shows coordinate expression of these 2 during chick hindlimb development (Sánchez-Ferrero et al. 2013). In addition, research on the rumpless Araucana breed proposed that ectopic expression of the *IRX1* and *IRX2* genes in the tailbud was the molecular driver for the lack of tail phenotype in Araucana (Freese et al. 2014). This conclusion was reached by exploring gene expression in embryos using in situ hybridization during HH stages 14 to 16 (50 to 56 h) (Freese et al. 2014). There, the hypothesis was that misexpression of *IRX* genes could potentially inhibit the expression of *TBX6*. However, our data suggest otherwise. In our findings, we observed that the expression of *TBX6* (Fig. 8E), along with *IRX1* and *IRX2* (supplementary fig. S2, Supplementary Material online), could be detected at later stages (E3.5 to E5.5, HH21 to 28) in WT samples. Importantly, we did not observe any repression between the expression of *IRX* genes and *TBX6* nor do we find any misexpression of *IRX1* and *IRX2* in Piao. Data from GEISHA (http://geisha.arizona.edu/geisha/) show that expression exist in tailbud at HH18 (E3) for *IRX1* and in mesoderm at HH17 (52 to 64 h) for *IRX2* in WT embryos was consistent with our finding that these genes are unlikely to be the functional drivers of the trait.

### Rum: a Novel Functional Candidate Gene to Regulate Tail Formation in Chicken

The novel *Rum* gene is located in a region containing multiple repeats, including 5 long interspersed nuclear elements (LINE) CR1,9 simple repeats, 3 low complexity repeats, and 1 DNA repeat element. Due to their common and widespread occurrence in the genome, both sequencing and primer design for exploring the region are challenging. Long-read RNA sequencing was used to explore the full-length expressed segment, but this did not provide information about the 2 ends of the *Rum* gene. Therefore “transcriptome walking” was used to reveal additional transcript information. However, the “walking” was unable to proceed past the “barrier”—repeats, and currently, the best estimate of the length of *Rum* is it being >22 kb containing no intron. Whether the *Rum* gene is a coding or noncoding gene is still uncertain, and our attempts to ascertain this were hampered by the lack of a full-length sequence. We conducted functional predictions and comparative genomics analyses to determine whether the *Rum* sequence shared homology with recognized proteins or functional domains. Unfortunately, we did not find any noteworthy similarities. Additionally, our predictions for long noncoding RNAs (lncRNAs) did not reveal any overlaps with the *Rum* gene. Its expression was only detected in embryos and not in those homozygous for the *Rp* rumpless allele. Expression was further reduced in heterozygous individuals (*Rprp*), normal in WT homozygous (*rprp*) birds. Our conclusion is that a single copy of *Rum* is unable to properly regulate the downstream target gene(s) crucial for tail somite formation resulting in the rumpless trait in Piao chicken.

### Time-Specific Differential Expression of MSGN1 and TBX6 Genes in Rumpless Embryos during Somite Segmentation

There was a significant differential expression of the *MSGN1* gene between Piao rumpless (*Rp* and *rp*) alleles. Significant expression was detected at E3.5 and barely afterwards in WT embryos. *MSGN1*, a helix–loop–helix (bHLH) transcription factor, function as 1 master regulator for differentiation and movement of presomitic mesoderm progenitor cells during development of somites in the tail (Fior et al. 2012; Yabe and Takada 2012; Chalamalasetty et al. 2014). Experiments from both mouse and zebrafish have demonstrated a key role of *MSGN1*, whose loss of function would downregulate the expression of *TBX6* in the presomitic mesoderm and lead to failure of somite formation and segmentation and the formation of an enlarged tailbud (Yoon and Wold 2000; Fior et al. 2012; Chalamalasetty et al. 2014; Manning and Kimelman 2015). This is consistent with our findings in that the expression of *MSGN1*, and *TBX6*, is significantly downregulated at E3.5 (Fig. 8C and E) in rumpless embryos. The expression of *MSGN1* markedly decreases at E4.5 in both *rprp* and *Rprp*, eventually disappearing (Fig. 8C). This indicates that the developmental regulation of somite segmentation by *MSGN1* is time-specific. The segmentation clock of vertebrates is controlled precisely till the last pairs of somites are reached (Gibb et al. 2010). The expression of the regulator *MSGN1* disappears once the segmentation process is complete, as a consequence of the tight temporal control of the segmentation clock. A similar mode of decreasing expression is observed for *TBX6* in both *rprp* and *Rprp* (Fig. 8E) at E3.5, followed by no significant differences between *RpRp* and *rprp* after somitogenesis is complete (Fig. 8A and E). Earlier studies have illustrated the combined function of these 2 genes in regulating presomitic mesoderm (PSM) differentiation (Wittler et al. 2007; Chalamalasetty et al. 2014; Manning and Kimelman 2015), while here, we demonstrate that *TBX6* might have a slightly different role in regulating tailbud development in chicken.

**Fig. 9.**
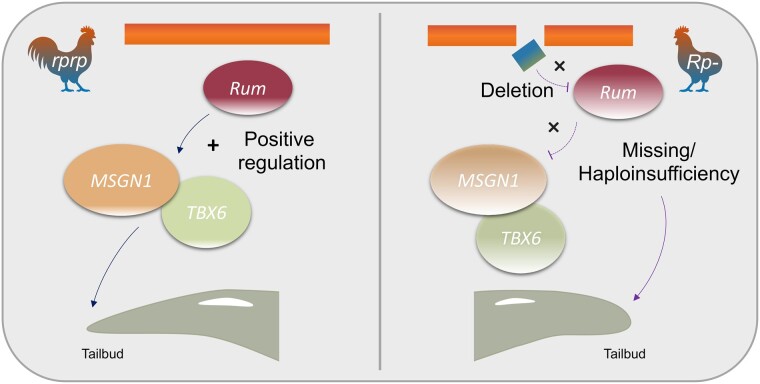
The molecular mechanism of *Rum* on regulating the rumpless trait in Piao.

Based on our results, we propose a model (Fig. 9) for tail formation whereby *Rum* acts as a positive regulator of *MSGN1* to activate the necessary factors required for mesoderm formation and differentiation. The deletion in the rumpless Piao allele results in a haploinsufficiency (*Rprp*) or completely missing (*RpRp*) of *Rum*, a dramatic downregulation of *MSGN1*, and functional related gene *TBX6* in mutant embryos at the crucial stage for the formation of tail somite. This downregulation ultimately leads to the development of the rumpless phenotype.

## Conclusions

A region on chromosome 2 was associated with the rumpless phenotype in the Chinese Piao chicken breed. Linkage analysis was used to fine-map the region to a <800 kb segment in a backcross population. Population and functional genomics analyses revealed that the rumpless phenotype was due to a different mutation than that earlier reported in the American Araucana breed. Whole-genome sequencing revealed a 4.2 kb deletion unique to Piao and completely linked to the rumpless trait in pure and crossbred Piao individuals. Functional genomics analyses identified a novel gene *Rum*, which was disrupted by the mutation. The resulting loss of *Rum* function by the deletion drives the ectopic regulation of the key regulator of somitogenesis via *MSGN1* and is concluded to be the molecular mechanism underlying the rumplessness trait in chicken.

## Materials and Methods

### Ethics Statements

This study was approved by the Animal Welfare Committee of China Agricultural University (approval number SKLAB-2013-0606), and all chickens used in the study were taken care of according to relevant regulations.

### Animal Material

#### A Piao × Xianju Backcross Reference Population

A backcross population (Table 1) was generated and maintained at the Jiangsu Institute of Poultry Science. First, *n* = 1 male Piao rumpless chicken was intercrossed with *n* = 4 female Xianju chickens with normal tails to produce the BC1 generation of *n* = 17 individuals. From these, a backcross population with *n* = 573 chickens in generations BC1 to BC6 was produced by repeated backcrossing of *n* = 1 rumpless Bc male to *n* = 8 to 10 Xianju females. Further, an intercross between rumpless BC1 chickens (BC1SM *n* = 1/4 males/females, respectively) was produced, and *n* = 9/25 normal/rumpless offspring were obtained. Individuals from this reference population were sampled for various uses throughout this study as detailed in Table 2.

**Table 2:** Details of the samples used from the Piao × Xianju backcross population

Experiment	Samples	Generation(s)	Sample type
SNP array genotyping	*n* = 16 rumpless (cases) *n* = 17 normal (controls)	BC1 and BC2	Blood/DNA
Whole-genome sequencing	*n* = 9 rumpless (cases) *n* = 3 normal (controls)	BC2 and BC6	Blood/DNA
Validation of deletion (PCR)	*n* = 163 rumpless (cases) *n* = 67 normal (controls)	BC1 to BC6	Blood/DNA
Embryonal somites	*n* = 106 random	BC3	Embryos E2.5 to E5.5

#### Purebred Normal and Rumpless Chicken Populations

The purebred Piao chicken population used in current study was sampled from the Jiangsu and Yunnan provinces, China. Individuals from this population were sampled for various uses throughout this study as detailed in Table 3.

**Table 3:** Details of samples obtained from purebred normal and rumpless chicken populations

Experiment	Sample(s)	Breed	Sample type
Embryonal somites	*n* = 56 random	Piao	Embryos E2.5 to E4.5
Whole-genome sequencing	*n* = 69 rumpless (cases) *n* = 7 normal (controls)	Piao	Blood/DNA
Validation of deletion (PCR)	*n* = 144 rumpless (cases) *n* = 10 normal (controls)	Piao	Blood/DNA

#### Public Sequencing Data

In the whole-genome sequencing–based analyses, also publicly available data from a range of populations were used as detailed in Table 4.

**Table 4:** Whole-genome sequence data obtained from public data

Analysis	Sample(s)	Breed	BioProject ID on NCBI
Genetic mapping	*n* = 5 rumpless *n* = 1 normal	Araucana	PRJNA524911 (Freese et al. 2014)
Population genetics	*n* = 81 normal	Black Minorca (*n* = 14) Langshan (*n* = 13) Dominique (*n* = 10) Black Cochin (*n* = 10) Buff Cochin (*n* = 9) Partridge Cochin (*n* = 4) Java (*n* = 10) Light Brahma (*n* = 21)	PRJNA552722 (Guo et al. 2019)

### Calculation of Embryonal Somites

Eggs were collected from both the reference backcross (*n* = 106) and purebred Piao (*n* = 51) as detailed in Tables 2 and 3. These were incubated at 37.5 °C until they reached the desired embryonal stages (E2.5 to E5.5; Table 5). Eggs were removed from the incubator, left to wait for 3 to 5 min to let the embryos come to the top of the yolk before cracking it into a Petri dish. A cyclic annular filter paper carrier was used to first cover the embryo. Next, a scissor was used to cut the vitelline membrane, before placing a second filter paper carrier on top of the first one and gently washing the embryo in phosphate-buffered saline (PBS) (37 °C) solution. Then, the embryo was carefully placed into the Petri dish to avoid bubbles before taking pictures using stereoscope (Nikon Ds-Fi1 and SMZ 1500) for subsequent counting of somites. Genotyping of the structural variant in embryos from E2.5 to E4.5 was done using PCR detection.

**Table 5:** Embryonal samples taken for calculation of somites

Embryonal stage	Population	Number of samples
E2.5	Piao BC3	*n* = 17 *n* = 18
E3	Piao BC3	*n* = 0 *n* = 15
E3.5	Piao BC3	*n* = 19 *n* = 22
E4.5	Piao BC3	*n* = 20 *n* = 16
E5	Piao BC3	*n* = 0 *n* = 18
E5.5	Piao BC3	*n* = 0 *n* = 17

### SNP Array, Genotyping, and Whole-Genome Sequence Data Processing

The first generations of the backcross family (BC1 to 2; *n* = 33; Table 1; Table 2) were genotyped using the Illumina chicken 60 K SNP array (Groenen et al. 2011). Quality control (QC) of the obtained genotypes was done using the following criteria: individual call rate >0.9, genotype call rate >0.9, and minor allele frequency (MAF) > 0.05. In total, *n* = 33 samples and 57,636 SNPs passed the (QC) and were used in the genetic analyses.

Additional individual SNP genotyping in the top association region from the GWAS analysis was initially performed using *Sequenom* (*n* = 55 SNPs) in 367 samples from Piao (*n* = 39), Xianju (*n* = 17), generations BC1 to 2 including BC1SM (*n* = 172), and other normal breeds (*n* = 139, 23 breeds) at an external company. Later, we transitioned to the *Fluidigm SNP Type* platform (*n* = 48 SNPs), set up within our own laboratory, in 593 samples from generations BC1 to 3 (*n* = 329), Piao (*n* = 136), Xianju (*n* = 36), and other normal chickens (*n* = 92, 15 breeds). This shift provided enhanced convenience and streamlined our experimental processes. The raw genotypes were filtered using the criteria (SNP call rate >80%, MAF > 1%), resulting in 21 and 13 SNP informative loci from the 2 assays (supplementary table S4, Supplementary Material online) to be used in the statistical genetic analyses.

Whole-genome sequencing was performed for *n* = 88 samples (Table 2, Table 3) on the Illumina HiSeq platform. The genome coverage for each sequenced individual varied from 2 to 5×. *GATK* (McKenna et al. 2010) was used for variant calling. The sequence reads were mapped to the GRCg6a/galGal6 reference using *BWA* (Li and Durbin 2009), and the alignment was processed using the *SAMtools* package (Li et al. 2009). *Picard* tools v2.20.4 (http://broadinstitute.github.io/picard/) was used for adding readgroups, sorting readpairs, marking duplicate reads, and building bam index. Local realignment, base quality score recalibration, and joint variant calling were implemented using the IndelRealigner, BaseRecalibrator, and HaplotypeCaller (*GATK v3.7*). QC filtering of the data was performed to only keep biallelic SNPs and to filter out low mapping quality SNPs where genotypes call rate <0.8, minor allele count <3, or minimum quality score <20 using *VCFtools* (Danecek et al. 2011). Phasing was then done on the filtered SNPs using *Beagle 4.0* (Browning and Browning 2007).

### Statistical Genetic Analyses

Association analyses were done using *Plink* (Chang et al. 2015) with “--assoc fisher -adjust” flags, and the results were plotted in R. *Plink* (Chang et al. 2015) was also used for IBD mapping in the BC1 to 2 using the 60 K SNP array data. *CRI-MAP* (Green et al. 1990) was used to estimate linkage between the individually genotyped SNP markers in the candidate association region detected in the GWAS analysis and the Piao rumpless phenotype using the 4 available families from BC2. The “prepare” option was used to create the other the .dat file and the .par file that are necessary for following analyses. The rumpless phenotype for each individual was considered as a marker, and the linkage with other markers was generated using “twopoint” option.

The population genetics selection scan analyses of the whole-genome sequence data were for Fst, Tajima’s *D*, and Pi diversity done using *VCFtools* (Danecek et al. 2011) and for haplotype differentiation using *hapFLK* (Fariello et al. 2013). All of these considered rumpless individuals as cases and normal individuals as controls.

### Scans for SVs in Sequence Data

Structural variant detection on chromosome 2 was done using *BreakDancer-1.3.6* (Fan et al. 2014). The configuration file was produced by the bam2cfg.pl script provided by the tools. The options used to detect the SV were “-a -q 20 -r 6 -o NC006089.5.” Fifty rumpless and 74 normal chickens were used for the analysis. The SVs with a total number of supporting read pairs that are <50 and not only identified in rumpless samples but also in WT samples are removed. After filtering, 5 SVs remained. One deletion (chromosome 2: 86,914,972 to 86,918,969 bp) was found in 45 rumpless samples with 183 read pairs supporting it (supplementary table S2, Supplementary Material online). The SV detection was also implemented in Araucana chickens with the if from PRJNA524911 (Noorai et al. 2019).

### Exploration of SV

Further PCR validation of the mutation was performed in the 5 rumpless samples where it was not detected in the whole-genome sequence data, and *n* = 384 additional samples (supplementary table S3, Supplementary Material online) showed that the deletion only existed and was present in all of the rumpless chicken. The samples included 154 Piao, 230 BC1 to 6, and 78 embryos (Tables 2 and 3). The primers used are given in supplementary table S6, Supplementary Material online. The PCR products were scored as containing the deletion or not using 1% agarose gel electrophoresis. Age estimation of the variation was inferred using *GEVA* (Albers and McVean 2020) based on the 798.5 kb sequence.

### Transcriptome Analysis

We utilized both full-length transcript (Pacbio RS II platform, *n* = 6) and short-read sequencing (Illumina NovaSeq 6000 platform, *n* = 18). This combination aimed to balance between comprehensive data acquisition and cost-effectiveness, ensuring a thorough and accurate representation of differentially expressed genes and transcripts at stages E3.5 and E4.5. RNeasy Micro kit (*QIAGEN*) was used for purification of total RNA from the tail tissue, and 14 µL RNase-free water was used for elution. Data analyses were performed using BMKCloud platform (https://www.biocloud.net/). Raw reads from Pacbio sequencing were processed using Iso-Seq pipeline with the criteria minFullPass = 3 and minPredictedAccuracy = 0.9. The high-quality full-length transcripts were filtered with the criteria: postcorrection accuracy >0.99. The short-read data from the Illumina platform was first processed in order to get high-quality data and aligned to an optimized GFF file derived from the long-read sequencing. Mapping was done using *Hisat2* (Kim et al. 2019). *Picard 1.41* (Broad Institute 2019) and *SAMtools 0.1.18* (Li et al. 2009) were used to sort, remove duplicates, and merge bam files. SNP calling was done using *GATK* (McKenna et al. 2010). The VCF files were filtered using the GATK standard filtering method. Differential expression analyses were performed using the *DESeq* (Anders and Huber 2012). Genes with an adjusted *P* value < 0.05 were classified as differentially expressed.

### Gene Expression Analyses

The expression of *IRX1* and *IRX2* was quantified by real-time qRT-PCR using *LightCycler 480* System. RNA was extracted from the tailbud of tailed (*rprp*, *n* = 13)/rumpless (*Rprp*, *n* = 19) embryos at days E3.5 (*n* = 4/5), E4.5 (*n* = 5/6), and E5.5 (*n* = 4/8) using RNeasy Micro kit (*QIAGEN*). Validation of the differentially expressed genes found from RNA-seq at days E4.5 (*n* = 9, 3 biological replicates/genotype) and E8.5 (*n* = 9, 3 biological replicates/genotype) was implemented using *Fluidigm Dynamic Array* which is a medium-throughput platform that can simultaneously detect the expression of 96 genes across 96 samples. For gene expression analysis that targeted a limited number of genes and individuals, the assessments were conducted on alternative low-throughput platforms, dictated by machine availability constraints. Expression of *TBXT*, *TBX6*, and *MEOX1* was detected at E3.5, E4.5, and E5.5, while expression of *MSGN1* was detected from E3.5 to E14 using *Applied Biosystems 7300* System. All 3 genotypes (3 biological replicates/genotype) were included except for days E5.5, E12, and E14, which only *rprp* and *RpRp* individuals were used. The genotypes of the embryos were scored using PCR of the SV type. cDNA was obtained from the extracted RNA by RT-PCR. The expression of each gene was normalized to the expression of *beta-actin*. The 2^−ΔΔCT^ method was used to calculate the relative changes in gene expression. The expression was normalized using the mean value from *rprp* of stage E3.5. *GraphPad Prism 6* (Swift 1997) was used for the statistical analysis. Figures were generated using R. The primers used for gene expression analyses are listed in supplementary table S6, Supplementary Material online.

## Supplementary Material

Supplementary material is available at *Molecular Biology and Evolution* online.

## Supplementary Material

msad273_Supplementary_Data

## Data Availability

The generated sequence data set is available on NCBI with the BioProject ID PRJNA961225.
