# Correction to: Mapping and Functional Dissection of the Rumpless Trait in Piao Chicken Identifies a Causal Loss of Function Mutation in the Novel Gene *Rum*

**DOI:** 10.1093/molbev/msae039

**Published:** 2024-03-29

This is a correction to: Ying Guo, Jing Tian, Chi Song, Wei Han, Chunhong Zhu, Huifang Li, Shuangjie Zhang, Kuanwei Chen, Ning Li, Örjan Carlborg, Xiaoxiang Hu, Mapping and Functional Dissection of the Rumpless Trait in Piao Chicken Identifies a Causal Loss of Function Mutation in the Novel Gene *Rum*, *Molecular Biology and Evolution*, Volume 40, Issue 12, December 2023, msad273, https://doi.org/10.1093/molbev/msad273

The originally published version of this manuscript required correction.

The authors identified an error in the correspondence of the values for Chi-square test presented in the section “Genetic Analyses of the Rumpless Trait”, under the subheading “Piao Rumpless Is an Autosomal Dominant Trait”. The following text contained the error:

The ratio of rumpless to normal birds was close to 1:1 in all backcross generations (Table 1), with no significant deviation from this when tested across all backcross chickens using a Chi-square test (χ2 = 0.10423; P value = 0.7468). The overall male:female ratio in the backcross population was also close to 1:1 (χ2 =0.29494, P value = 0.5871).

This has been corrected to read as follows:

The ratio of rumpless to normal birds was close to 1:1 in all backcross generations (Table 1), with no significant deviation from this when tested across all backcross chickens using a Chi-square test (χ2 = 0.29494; P value = 0.5871). The overall male:female ratio in the backcross population was also close to 1:1 (χ2 = 1.4677, P value = 0.2257).

In addition, Figure 2E has been updated to correct the placement of the red triangle.

