# Supplementary material for: Mapping and Functional Dissection of the Rumpless Trait in Piao Chicken Identifies a Causal Loss of Function Mutation in the Novel Gene Rum
Source: Mol Biol Evol. 2023 Dec 9;40(12):msad273. doi: 10.1093/molbev/msad273 (PMC10735294; doi:10.1093/molbev/msad273)

**Supplementary files**

**Figure S1. Multiple alignment of the deletion sequence in 77 species.** The figure from UCSC shows the deletion sequence alignment in 55 birds (red box), 10 reptiles (yellow box) and 12 other vertebrate species (blue box) including human, mouse and frog etc.


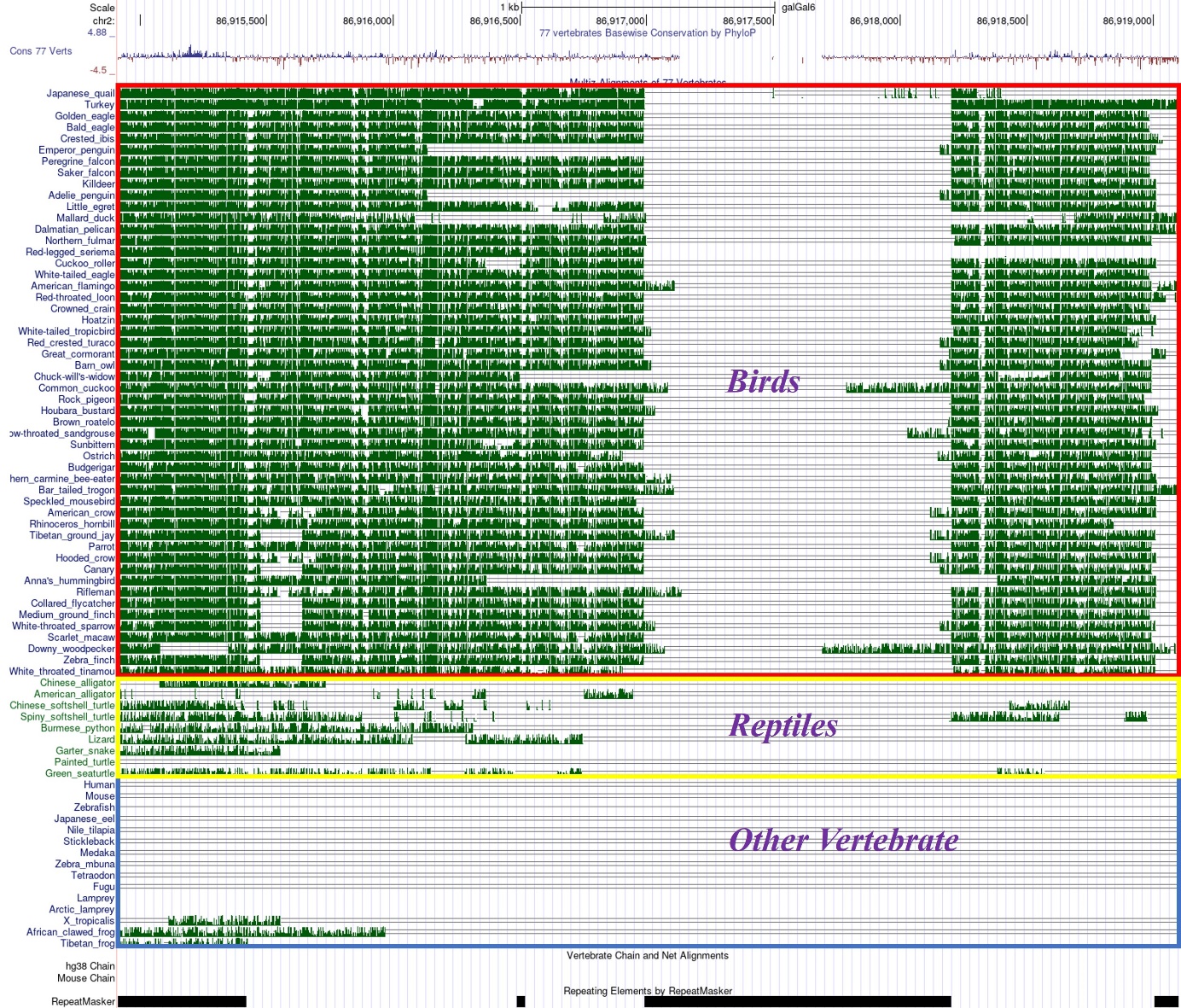


**Figure S2. Detection of genes expression.** The relative expression of *IRX1* (A) and *IRX2* (B) at E3.5, E4.5 and E5.5 in both wild-type and rumpless embryos. No significant difference was identified.


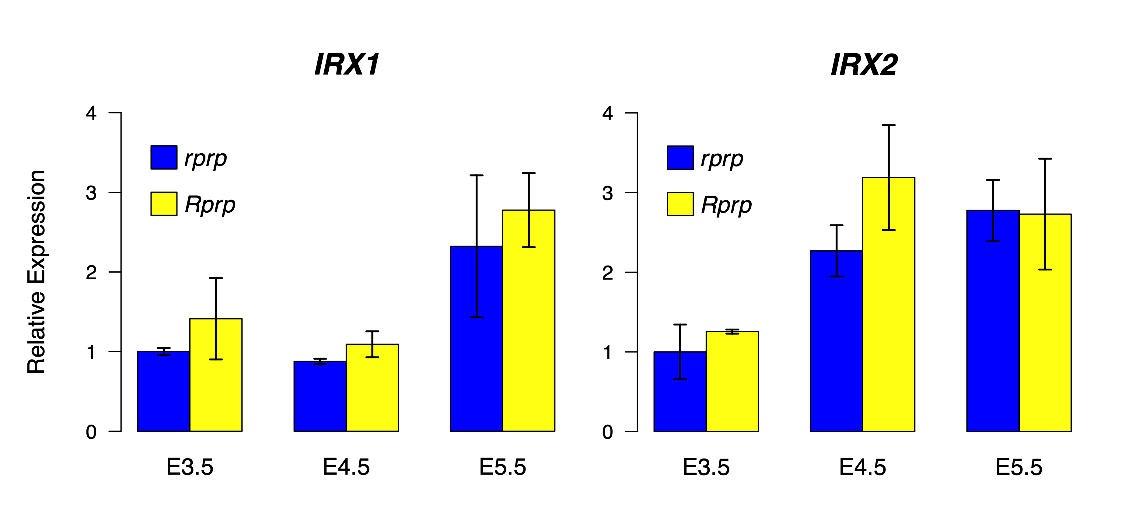


**Figure S3. “Transcriptome Walking” of the novel transcript.** Overlapped RT-PCR analyses of both ends for the predicted ORF proved that the length of *Rum* is at least 22270 bp.


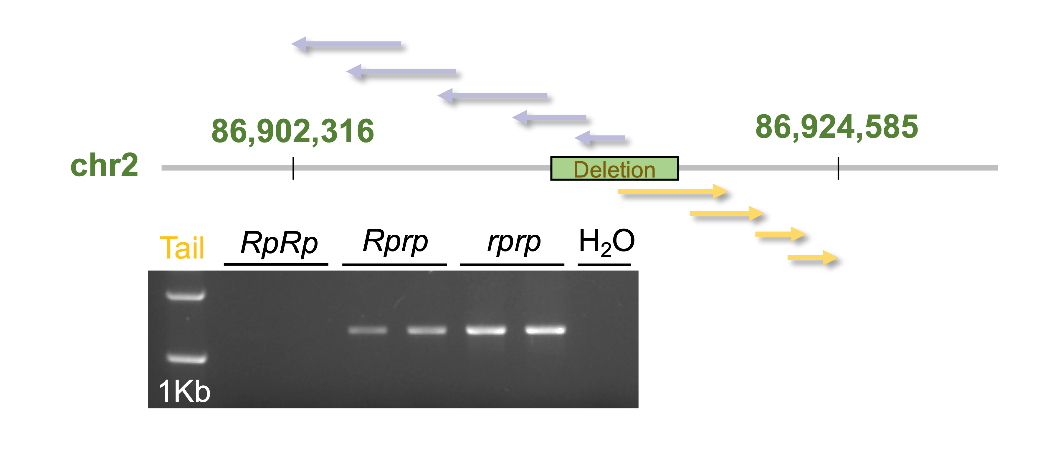


**Figure S4. Detection of the expression of *MSGN1*.** The expression of both the common and long-specific regions were detected using RT-PCR (A and B). The relative expression of the common region of *MSGN1* was evaluated at different developmental stages. ****p* < 0.001, ***p* < 0.01, **p* < 0.05.


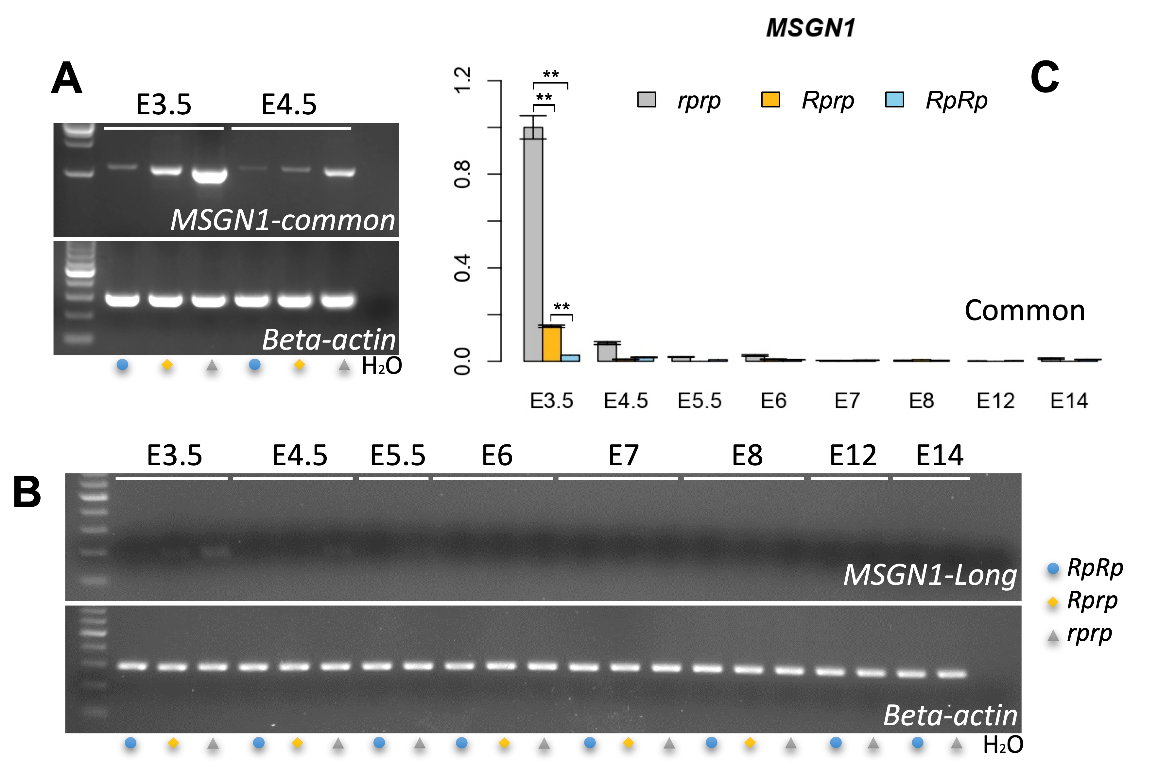

Supplement: msad273_Supplementary_Data [file msad273_supplementary_data.zip › Supplementary_Files.docx]
