# Supplementary figures and images for: Mapping and Functional Dissection of the Rumpless Trait in Piao Chicken Identifies a Causal Loss of Function Mutation in the Novel Gene Rum
Source: Mol Biol Evol. 2023 Dec 9;40(12):msad273. doi: 10.1093/molbev/msad273 (PMC10735294; doi:10.1093/molbev/msad273)

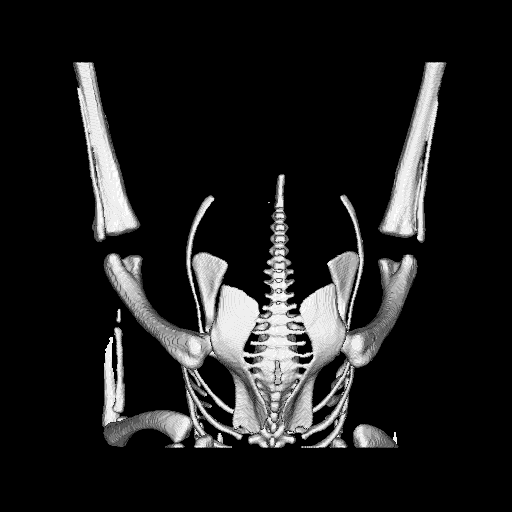

Supplement: msad273_Supplementary_Data [file msad273_supplementary_data.zip › Wildtype.gif]

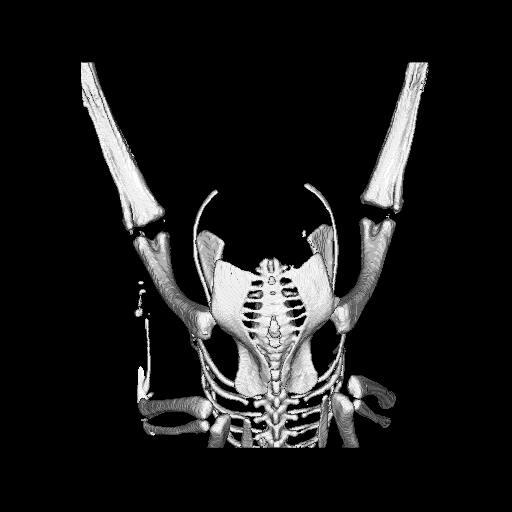

Supplement: msad273_Supplementary_Data [file msad273_supplementary_data.zip › Rumplessness.gif]
